# Cerebral lateralisation during signed and spoken language production in children born deaf

**DOI:** 10.1016/j.dcn.2019.100619

**Published:** 2019-01-24

**Authors:** Heather Payne, Eva Gutierrez-Sigut, Bencie Woll, Mairéad MacSweeney

**Affiliations:** a Deafness, Cognition & Language Research Centre, University College London, WC1H 0PD, UK; b Institute of Cognitive Neuroscience, University College London, WC1N 3AZ, UK; c Departamento de Metodología de las Ciencias del Comportamiento, Universitat de València, Av. Blasco Ibáñez, 2146010, Spain

**Keywords:** Language, Lateralisation, Functional transcranial Doppler sonography, fTCD, Deaf, Children, Cochlear implants, Sign language

## Abstract

The effect of sensory experience on hemispheric specialisation for language production is not well understood. Children born deaf, including those who have cochlear implants, have drastically different perceptual experiences of language than their hearing peers. Using functional transcranial Doppler sonography (fTCD), we measured lateralisation during language production in a heterogeneous group of 19 deaf children and in 19 hearing children, matched on language ability. In children born deaf, we observed significant left lateralisation during language production (British Sign Language, spoken English, or a combination of languages). There was no difference in the strength of lateralisation between deaf and hearing groups. Comparable proportions of children were categorised as left-, right-, or not significantly-lateralised in each group. Moreover, an exploratory subgroup analysis showed no significant difference in lateralisation between deaf children with cochlear implants and those without. These data suggest that the processes underpinning language production remain robustly left lateralised regardless of sensory language experience.

## Highlights

• Children born deaf show typical left-hemisphere dominance during language production.
• No evidence of an association between left-lateralisation and language proficiency.
• Exposure to auditory speech via a cochlear implant is not a prerequisite for left hemisphere language dominance.

## 1 Introduction

Language processing in the vast majority of hearing adults is associated with more extensive brain activity in the left than right hemisphere (Knecht et al., 2000; Price, 2012; Vigneau et al., 2006). Asymmetric patterns of activity are also found in children producing language (Bishop et al., 2009; Krishnan et al., 2015; Paquette et al., 2015; Sowman et al., 2014). The causal underpinnings of hemispheric specialisation for language are not known, but are likely to involve a complex interaction of genetic, maturational, hormonal, and experiential factors (Bishop, 2013; Francks, 2015; Josse and Tzourio-Mazoyer, 2004; Tzourio-Mazoyer et al., 2016). One such factor is sensory experience, manipulation of which has been shown to influence hemispheric lateralisation of visual processing in other animals (Johnston and Rogers, 1999; Letzner et al., 2014; Ocklenburg and Güntürkün, 2012). In humans, manipulating early sensory experience is not ethical. We can however observe the effect of altered sensory experience on lateralisation by examining cerebral lateralisation in individuals who have sensory impairment. One such example is children born deaf.

Children born deaf show great variability in their experience of language. First, their auditory language input is variable. This is largely dependent on the child’s level of deafness and whether they receive a device such as a hearing aid or cochlear implant (CI), which may or may not provide some useful access to auditory speech. Second, deaf children’s visual language experience is also variable. For all deaf children, access to spoken language is supported by visual input (lipreading). In addition, 5–10% of deaf children may be exposed to a signed language at home from their deaf parents. Others may learn sign language at some point during their school years or later in life, while others still might never learn sign language.

In a review of the literature on the development of hemispheric lateralisation, (Minagawa-Kawai et al., 2011), delineate three types of theory that aim to account for asymmetries in brain responses to speech. Although the focus of the review is speech perception, it nevertheless provides a useful framework against which to consider lateralisation for other domains of language processing. The three types of account are summarised as signal-driven, domain-driven, or those which emphasise the role of learning.

Signal-driven theories focus on low-level properties of speech and their differential processing in early auditory areas (Boemio et al., 2005; Giraud et al., 2007; Morillon et al., 2010; Poeppel, 2003; Zatorre and Belin, 2001). For example, temporal or spectral complexity, optimal time windows for integration, and phoneme or word duration have all been put forward as candidate properties for inducing different processing streams. Evidence for a signal-driven account comes from findings of left or right lateralised brain responses in infants in auditory cortices and neighbouring portions of the superior temporal cortex, depending on the specific acoustic properties of the stimuli (for example, Dehaene-Lambertz et al., 2002; Peña et al., 2003; see Minagawa-Kawai et al., 2011 for a review). Whether experience of auditory input to these regions plays a causal role in lateralisation of later language production is not known, but this possibility has been raised in the literature (for example, Poeppel, 2012).

Domain-driven accounts suggest that abstract linguistic features drive asymmetries in processing. These accounts place emphasis on a left-lateralised brain network that processes linguistic information regardless of the properties of the sensory input (Dehaene-Lambertz et al., 2006). Previous findings of robustly left-lateralised activity in deaf adult deaf signers processing sign language provide support for this view (Emmorey et al., 2002a; Gutierrez-Sigut et al., 2016; MacSweeney et al., 2002, 2008). The importance of linguistic information is also evident from studies in which the low-level properties of the stimulus remain constant, but task is manipulated to involve more or less linguistic processing. For example, left lateralisation has been demonstrated to emerge only after hearing participants with no knowledge of a sign language are given labels for a set of gestures (Möttönen et al., 2010) or when participants are required to focus on certain aspects of an auditory speech stimulus such as meaning versus tonal quality (Von Kriegstein et al., 2003).

The third type of theory to account for hemispheric lateralisation of language attributes a critical role to learning. Support for this perspective comes from studies of songbirds demonstrating increasing left-lateralisation with song learning (Chirathivat et al., 2015; Moorman et al., 2015; Tsoi et al., 2014). Significant asymmetry (left > right) in the formation of new neurons in auditory regions is reported for zebra finches (Tsoi et al., 2014). Human adults also show increased functional left lateralisation as they learn a foreign language (Plante et al., 2015). At the other end of the spectrum, there is some evidence that individuals with language learning disorders show atypical lateralisation. Reduced functional lateralisation has been reported for language impaired groups (Artiges et al., 2000; Braun et al., 1997; Crow et al., 1996; de Guibert et al., 2010; Maisog et al., 2008; Whitehouse and Bishop, 2008) though some of these findings are subject to controversy over their validity (see Wilson and Bishop, 2018).

Contrasting patterns of lateralisation in deaf and hearing children can provide insights into these different accounts of hemispheric lateralisation for language. A signal-driven account of lateralisation predicts that children born deaf are more likely to show atypical cerebral lateralisation, or to show ‘weaker’ lateralisation, for language, as a consequence of reduced access to auditory speech. In contrast, a domain-driven account predicts deaf children will show left-lateralisation regardless of modality of language background, as long as they are exposed to complex linguistic information. A learning-based account also predicts left-lateralisation regardless of language background, but crucially only in those individuals with proficient language.

To date, only one study has investigated functional hemispheric asymmetries in children born profoundly deaf. Chilosi et al. (2014) reported group-level left lateralisation in deaf children. This was interpreted as evidence for the importance of auditory experience, provided via a cochlear implant (CI), in the development of left lateralisation. However, *only* deaf children who had an early CI were included in the study. Therefore it is not possible to draw conclusions regarding the relationships between auditory input via a CI and lateralisation for language. A better test of the signal driven account would be to assess hemispheric lateralisation in a more representative sample of deaf children, some of whom do not have CI. This is the approach taken in the current study.

Investigating the neural systems involved in language processing in deaf children has been hampered by the incompatibility of CIs with common forms of functional imaging, such as fMRI. In the current study we use functional transcranial Doppler sonography (fTCD), which assesses gross differences in hemispheric activity (Deppe, Ringelstein, & Knecht, 2004). fTCD, the method also used by Chilosi et al. (2014) measures changes in the speed of blood flow through the left and right middle cerebral arteries (MCAs) during sensory and cognitive tasks (Aaslid et al., 1982). Two features of fTCD make it ideal for assessing language lateralisation in deaf children. First, fTCD is not subject to the same constraints on movement as other imaging modalities, permitting the measurement of cerebral blood flow changes during overt speech or sign language production (Emmorey et al., 2002b; Gutierrez-Sigut, Payne, & MacSweeney, 2016; MacSweeney et al., 2002, 2008). Second, fTCD is safe for use with those with cochlear implants, which are contraindicated for use with fMRI.

In the current study, we recruited a diverse group of young deaf children and measured lateralisation during language production in their preferred communication mode. As well as variability in language input, deaf children’s language output also varies from child to child and within a child, depending on the context and the interlocutor. It is not uncommon for a deaf child to switch between spoken English and BSL, or to use a form of communication with the grammar of spoken English, with accompanying BSL signs (often referred to as Sign Supported English or Total Communication). We compared the lateralisation strength in deaf children during language production in their preferred communication mode, with that of language-matched hearing comparison group. Offline behavioural measures of language proficiency were also collected so that we could investigate the relationship between lateralisation and language proficiency.

With regard to the effect of cochlear implantation on lateralisation, support for the conclusions drawn by Chilosi et al. would come from evidence showing that deaf children with CI (who have more experience with auditory speech) show greater left lateralisation than those without. This study was not powered to detect differences between amplification types, therefore this comparison is included as exploratory.

In contrast to Chilosi et al. (2014) we suggest that exposure to auditory speech may not be the main driving factor in the establishment of left lateralisation. We suggest that learning- or domain-driven theories of lateralisation are also tenable. Therefore, we predicted that a heterogeneous group of deaf children would show left lateralisation during language production. We predicted a similar strength of lateralisation in the deaf and hearing children, since the groups were matched on language level. In line with the idea that lateralisation is related to an efficient language processing system, we predicted a positive relationship between strength of lateralisation and language ability, irrespective of hearing status.

## 2 Method

### 2.1 Procedure

Ethical approval for the study was obtained from the UCL Research Ethics Committee. Parents or carers gave written informed consent prior to the study. Children also gave verbal assent before the start of each testing session.

Testing took place over two or three sessions on separate days not more than a week apart. Children were tested in a designated room at their school by a hearing researcher or a deaf researcher fluent in BSL. In some cases, a learning support assistant accompanied the child. All children were encouraged to respond in whichever language (or combination of languages) they wished. Children who communicated predominantly in BSL were given all instructions in BSL.

### 2.2 Background measures

#### 2.2.1 Cognitive assessments

For children in both groups, nonverbal IQ was assessed using the Pattern Construction subtest of the British Ability Scales, 3^rd^ edition (BAS-III; Elliot and Smith (2011)).

#### 2.2.2 Language assessments

Due to differences in ages and language modalities across and within groups, it was not possible to use the same language assessments for all participants.

##### 2.2.2.1 Language assessments for deaf children

In the deaf group, general expressive vocabulary knowledge was estimated using the Naming Vocabulary subtest of the British Ability Scales (BAS-III; Elliot & Smith, 2012). Given that standard scores for these scales are normed for hearing children, we report scaled scores which take into account the number of attempted items. Children were able to respond in either English or BSL.

We also included assessments of receptive language in both English and BSL, where appropriate. The Single Words subtest of the Test of Child Speechreading (Kyle et al., 2013) was included as a measure of spoken English language comprehension. British Sign Language (BSL) comprehension was assessed using the BSL Receptive Skills Test (BSL-RCT, Herman et al., 1999), which focuses on receptive grammar. Children were only tested on this assessment if 1) their teacher reported the child having exposure to BSL and 2) they could produce 50% or more of the BSL labels for the nouns that appeared in the test. Using these criteria, 6 out of 19 children were tested on the BSL RCT.

Finally, the Early and Single Word Reading subtests of the York Assessment of Reading Comprehension (YARC, Hulme et al., 2009) were included as additional measures of language proficiency. Single word reading was attempted only if the child could complete the majority of the Early Word Reading subtest. Children were able to respond in either English or BSL.

##### 2.2.2.2 Language assessments for hearing children

For the hearing group, expressive vocabulary was assessed using age appropriate subtests of the BAS-III (Elliot & Smith, 2012). Four to five year olds were tested on the Naming Vocabulary subtest and 6–7 year olds were tested on the Word Definitions subtest. We did not include any measures tapping receptive language skills.

We also assessed children on the Early/Single Word Reading subtests of the YARC (YARC, Hulme et al., 2009). Single word reading was attempted only if the child could complete the majority of the Early Word Reading subtest.

#### 2.2.3 Handedness assessments

Handedness was assessed using two measures of hand preference. The card-reaching task followed the procedure described by Bishop et al. (1996). A set of cards depicting highly nameable objects were dealt into 7 piles at 30 ° intervals in a semi-circle in front of the child. In a random order, which was the same for every child, the experimenter asked the child to reach for a given object card and place it in a pile in front of them. A Card Laterality Quotient (Card LQ) was calculated for each child, given by LQ = (R − L) / (R + L + Both) *100, ranging from 100 for participants reaching exclusively with the right hand, 0 for those who do not show a preference, to −100 for those reaching exclusively with their left hand.

The second measure of hand preference required children to use four objects in turn: a pencil, scissors, a jug, and a cup. An Objects Laterality Quotient (Object LQ) was calculated in the same way the Card LQ, given by LQ = (R − L) / (R + L + Both) * 100.

### 2.3 Participants

Twenty-eight deaf (greater than 60 dB loss in their better ear) children were recruited from UK hearing support units (16), specialist deaf schools (11) and mainstream schools (1). It was not possible to record fTCD data for six of these children due to inability to find a signal (4 children), interference with the placement of probes from glasses (1 child), or inability to attempt a sufficient number of trials (1 child). Therefore, data were collected from 22 children (13 male). The average age of the sample was 8; 0 in years; months (min = 5; 0, max = 11; 5). All children had typical non-verbal IQ (mean standard score = 49.4, min = 34, max = 66).

For comparison, we included a group of hearing children who were tested as part of another study (Payne et al., in prep). The average age of the comparison group was 6; 4, (min = 4; 3, max = 7; 6). These children also had typical non-verbal IQ scores (mean standard score = 58.6, min = 47, max = 78). The group was selected on a case-by-case basis according to their single word reading ability to be closely matched to the deaf group. Reading scores were chosen a proxy for language ability since this test was used with the majority of both deaf and hearing children. Three deaf children had especially low language ability. Three younger hearing children were selected to be their matched controls, who had similar vocabulary naming age equivalents (Naming Vocabulary subtest of the BAS-III) and Early Word Reading scores. These six children were not included in correlations relating reading score with LI. Children in the hearing group were significantly younger than those in the deaf group (t (36) = 3.30, p = .002) but not significantly different in terms of reading scores (t (28) = 0.05, p = .96) or vocabulary (t (4) = 0.27, p = .79).

The deaf and hearing groups were also matched on two measures of hand preference, tool use and card reaching, as described in Section 2.2.3. The majority of children showed strong right hand dominance in these tasks. The groups did not differ on either of these measures (Mean Object LQ deaf = 81.6 (sd = 41.5), hearing = 77.6 (sd = 47.8), t (36) = .27, p .79, Mean Reaching LQ deaf = 49.5 (sd = 48.3), hearing = 36.9 (sd = 37.0, t (34) = .88, p = .38). Three participants in the deaf group and one participant in the hearing group wrote with their left hand. All four of these participants are denoted by open circles in the Results shown in Fig. 3.

### 2.4 fTCD materials

Blood flow velocity through the left and right MCAs was measured using a Doppler ultrasound device (DWL DopplerBox: manufactured by DWL Elektronische Systeme, Singen, Germany). Two 2-MHz transducer probes were mounted on a flexible headset and placed at each temporal skull window. To accommodate cochlear implants and hearing aids, we commissioned a custom-built band headset to hold the ultrasound probes in place.

Changes in cerebral blood flow velocity (CBFV) were recorded during an Animation Description task developed in Bishop et al. (2009) which has been used with children as young as 4-years-old and also adults (Bishop et al., 2009; Groen et al., 2013, 2012; Hodgson et al., 2016) and shows good reliability within and across testing sessions (Bishop et al., 2009).

**Fig. 1:**
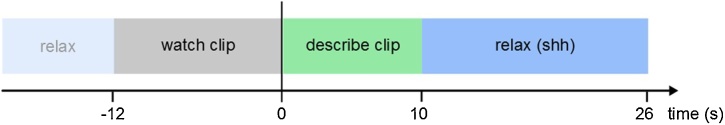
Schematic showing timings for single trial of Animation Description.

During the task, the child watched a cartoon penguin in a series of clips. The maximum number of possible clips was 30. However, this was dependent on the child’s compliance. The animation had environmental sounds and some unintelligible vocalisations but was otherwise silent. Fig. 1 shows a schematic of the trial timings. A period of silent watching (12 s) was followed by a prompt for the child to describe the events of the animation (10 s) after which the child was instructed to sit quietly for a rest period of 16 s. After the rest period, the experimenter checked the child is ready to proceed. The task was presented using Cogent toolbox (www.vislab.ucl.ac.uk/cogent) for MATLAB (Mathworks Inc., Sherborn, MA, USA). Triggers were time-locked to the onset of the video clips.

### 2.5 Planned analyses

#### 2.5.1 Behavioural data processing

Due to the very different language outputs of the deaf and hearing children, different measures of amount of language produced during the task were established for the two groups. For the hearing group, responses were audio recorded and transcribed offline. As an indication of task performance, the average number of words produced for accepted trials was calculated. For the deaf group, responses were recorded using a digital video camera. Videos of behavioural responses were coded using ELAN transcription software (http://tla.mpi.nl/tools/tla-tools/elan; Max Planck Institute for Psycholinguistics, The Language Archive, Nijmegen, The Netherlands). This allows time-aligned annotations for multiple tiers of description. For example, a child’s utterances can be marked using a separate tier for each hand and another tier for speech. It was not possible to code the number of tokens produced (words or signs) because deaf children often switched between spoken English, BSL, and gesture or produced these outputs at the same time. In some of the youngest children particularly it was difficult to differentiate poorly-formed signs from gestures to get an accurate measure of the number of individual tokens. Instead we coded onsets and offsets of the following actions separately for right-hand, left-hand, and speech: during the video presentation (the period used for baseline correction), during the animation description period and during the ‘relax’ period. Total seconds of each transcription were calculated and averaged over the number of viable trials for that child. From the transcriptions, we calculated an index of hand-movement dominance: (R – L) / (all hand movement) * 100 as well as the average communication (of any type) for each child. Instances where the child produced utterances during the baseline or ‘relax’ period were manually rejected. Instances of self-grooming, brief points or single short vocalisations were ignored.

#### 2.5.2 fTCD data processing

Analyses were run using functions from https://github.com/nicalbee based on dopOSCCI, a custom MATLAB (Mathworks Inc., Sherborn, AM, USA) toolbox written for analysing fTCD group data (Badcock et al., 2018; Badcock, Holt, Holden, & Bishop, 2012). The analysis scripts used in the current study are available at from the OSF page for this project - https://osf.io/r3evy/?view_only = 9350d5137f024804bc06b42c3672bae6.

Analyses of the cerebral blood flow velocities consisted of normalisation of left and right channel values on a trial-by-trial basis and integration of blood flow speed fluctuations associated with the heart cycle (Deppe et al., 1997). In addition, trials with unusually high or low blood flow speeds were rejected. This was defined as ±50% of the average speed for that channel.

The data were segmented into epochs from –12 to 26 s relative to stimulus presentation. The left-right difference wave was baseline corrected by subtracting the average blood flow velocity during a period of inactivity (10 to −2 s prior to stimulus onset) from each data point. As is standard for fTCD analysis, a period of interest (POI) was chosen *a priori*. Here the POI was set between 4 and 18 s.

For each participant, the maximum peak left-right difference within the POI was identified. A two second window was centred on this maximum. The LI was defined as the average of the left minus right difference within this two second window. One-sample t-tests were used to assess whether the LI value was significantly left or right lateralised for each participant in each condition. When one-sample t-tests were not significant, participants were considered ‘low lateralised’. The group mean LI was calculated as the mean average of individual LIs.

## Results

### 3.1 Behavioural data

Descriptive statistics for language assessments are presented in Table 1. With regard to amount of linguistic material produced by deaf and hearing children during the tasks, equivalent measures could not be established (as described in Section 2.5.1). We include descriptive statistics here only to provide a better description of the variability within each group, not for comparison. Deaf children produced an average of 7.8 s of communicative content (in any form) per 10 s active period (sd = 2.3, range = 1.5–10.63). Hearing children produced an average of 17.3 words per 10 s active period (sd = 2.8, range = 12–23).

**Table 1:** Descriptive statistics for language assessments and online task performance. Group comparisons are made where appropriate.

	Deaf	Hearing	Test statistic	*p*	Effect size
	Mean (SD)				
Age	8.19 (2.17)	6.38 (1.01)	t(36) = 3.30	.002	1.1
Test of Child Speechreading Single words (%)	70.7^†^ (8.0)	–	–	–	–
BSL Receptive Skills^†^	84.0 (13.5)	–	–	–	–
Expressive vocabulary Naming^§^	n = 16	–	–	–	–
	161.3 (24.0)				
Expressive vocabulary	–	n = 16	–	–	–
Word Definitions^§^		100.9 (16.5)			
Single word reading^‡^	31.07 (12.90)	31.31 (11.92)	t (28) = .053	.958	0.009
Participants with low language proficiency					
Early word reading^‡^	n = 3	n = 3	t(4) = .18	.87	0.15
	3.0 (4.4)	3.7 (4.6)			
Expressive vocab	n = 3	n = 3	t(4) = 1.9	.13	1.5
Naming^‡^	16.7 (4.5)	23.3 (4.0)			

a Corresponds to scores between 50^th^ – 75^th^ centile.

† standard score; ^‡^ raw score; ^§^ scaled ability score (takes into account number of items attempted).

### 3.2 fTCD data quality and reliability

Data from two children were identified as poor quality during the first phase of data analysis. Following artefact rejection, these two children had a low number of acceptable trials (<9) and were excluded from further analyses. This threshold for the number of acceptable epochs is within the range commonly used for fTCD studies (Badcock and Groen, 2017).

In the second stage of data screening, trials were removed if the child had not adhered to the instructions. For one child this resulted in fewer than 9 acceptable trials and they were excluded from further analyses. The statistics below reflect the data from the more restricted group of 19 deaf children (10 male) and 19 hearing children (9 male). The average age of this group was 8; 0 years (min = 5; 0, max = 11; 5).

Reliability was estimated using the split-half correlation coefficient for the averaged odd and even LIs calculated for each individual. Reliability for both groups was good (deaf: r = 0.91, p < .001, hearing: r = .82, p < .001). This indicates a consistent response to the task across the trials. The average number of suitable trials was 14.4 (sd = 3.8, min = 9, max = 22) for the deaf group, and 14.6 (sd = 2.6, min = 10, max = 19) for the hearing group. The average latency of the peak difference within the period of interest was 10.9 s (sd = 2.9, min = 6.4, max = 17.7) for the deaf group, and 11.2 s (sd = 2.9, min = 6.0, max = 17.7) for the hearing group. There were no significant differences between groups on any of these measures (all p’s > 0.1)

### 3.3 Group lateralisation indices

**Fig. 2:**
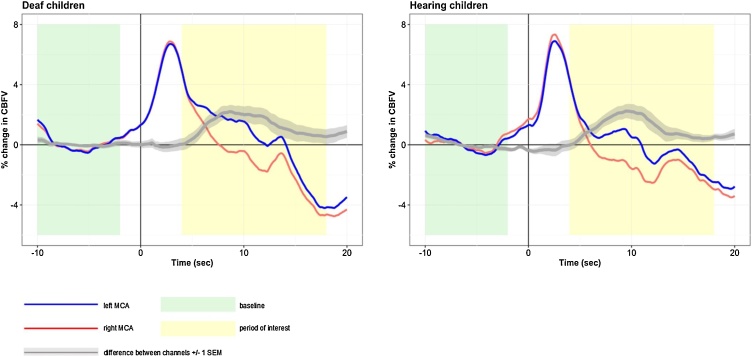
Average blood flow velocity change in left and right Middle Cerebral Arteries (MCA) during Animation Description for children born deaf (left side) and hearing children (right side).

The average LI of the deaf group was 2.2 (3.0), indicating significant left lateralisation (one sample t-test, t (18) = 3.2, p = .005). The average LI of the hearing group was 1.8 (3.0), which is significantly different from 0 (t (18) = 2.7, p = .016) indicating left lateralisation at the group level. Table 2 shows descriptive and inferential statistics for the LIs of both groups, as well as the percentage of children in hearing and deaf groups categorised as left- right- or low-lateralised according to their individual LI and SEM. Fig. 2 shows both groups’ averaged time course of cerebral blood flow velocity change for left and right channels and the average difference between channels.

**Table 2:** Descriptive statistics for fTCD data and group contrasts for deaf and hearing children.

	Deaf	Hearing	Test statistic	*p*	Effect size (Cohen’s d)
N	19	19			
No. trials	14.3 (3.7)	14.6 (2.6)	t(36) = .25	.80	.09
LI	2.23 (3.02)	1.79 (2.95)	t(36) = .45	.66	.15
N left (%)	15 (79)	13 (68)	χ²(2) = .62	.73	
N right (%)	3 (16)	4 (21)			
N low (%)	1 (5)	2 (11)			
Peak latency (seconds)	10.9 (2.9)	11.2 (2.9)	t(36) = 0.29	.78	.10
Odd/even reliability	.91	.82	Test on fisher transformed z = 1.05	.15

**Fig. 3:**
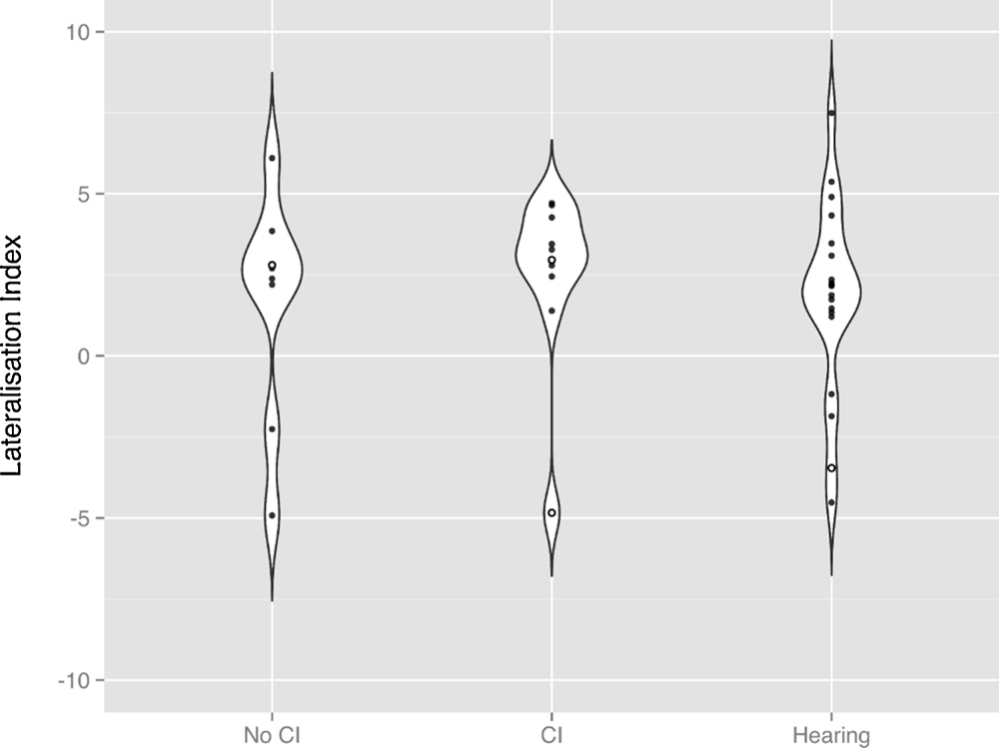
Violin plots showing the estimated probability density function of the LI distribution for each group. Open circles denote left-handed individuals.

We compared the strength of lateralisation between the deaf and hearing groups. There were no significant differences between the hearing and deaf groups in terms of strength of LI (t (36) = .45, p = .67, d = 0.15). Furthermore, there were no significant differences in the proportion of children in each lateralisation category (χ² = .62, p = .73). See Table 2.

### 3.4 Effect of implant status (CI n = 10, non-CI n = 8)

As a further exploratory test of whether access to auditory speech through a CI plays a role in the development of lateralisation for language processing, we compared the strength of lateralisation between deaf children who had or had not received a cochlear implant. One child was not included in this analysis because they had received a Bone Anchored Hearing Aid (BAHA). These devices use bone conduction to transmit sound to the inner ear. Thus, they are very different amplification devices to CIs. Furthermore, it would be problematic to include a child with a BAHA in a ‘no CI’ group because the assumption is that children in a ‘no-CI’ group would have reduced transfer of auditory information to the cochlea. There was no significant difference in average LI between the subgroups: CI group = 2.5 (sd = 2.8); non-CI group = 1.6 (sd = 3.5) (t (16) = .61, *p* =  .55, d = 0.28). Fig. 3 shows the individual LIs for children in the no-CI, CI, and hearing group respectively.

### 3.5 Relationship between language ability and lateralisation

To test the hypothesis that lateralisation is driven by learning, we tested the relationship between the strength of lateralisation and an offline behavioural measure of language proficiency for all children, regardless of hearing status. The correlation between single word reading and strength of lateralisation was not significant either when all participants were combined (r = .24, p = .20) or when each group was tested separately (deaf: n = 14, r = .16, p = .58, hearing: n = 16, r = .32, p = .23).

### 3.6 Test of equivalence

As described in Section 3.3, we did not observe a significant difference in the strength of lateralisation between deaf and hearing groups. This null result may reflect a true difference of 0 between groups, or may be due to insufficient power to detect a given effect size. In the latter case, judgement on the existence of a group difference should be reserved until more data have been collected. To test whether we can be confident in this null result, we ran a test of equivalence on the difference in LI between deaf and hearing groups, alongside calculating a Bayes factor for the likelihood of the null over the alternative (observed) distribution. This was done using the Two One-Sided T-tests (TOST) library for R, as described in (Lakens et al., 2018; Lakens, 2017).

For these procedures, it is necessary to specify several parameters. For the TOST procedure, a smallest effect size of interest (SESOI) is selected *a priori*. A group difference with an effect size within these bounds suggests that the groups can be considered equivalent. One approach to defining a smallest effect size of interest is to calculate a critical test statistic for a previous study that tested the same hypothesis. In adopting this logic, we accept that there may be smaller true differences between groups but elect that they are small enough to be theoretically non-informative. Chilosi et al. (2014) tested 40 children with an α=0.05. A critical t-value for this study would have been 2.02, which is equivalent to a critical Cohen’s d=0.64. Thus, any group difference of less than 1.9 units (in raw measurement of change in blood flow velocity) would not reach significance. This value is taken as the SESOI for the test of equivalence on our data.

For the comparison of Bayes factors we use an informed prior distribution also based on the findings of Chilosi et al. (2014), and our *a priori* hypothesis (that deaf children will be less strongly left-lateralised than hearing children). The consequence of this is modelling the prior as B_H(0,0.5)_. The subscript _H_ refers to a half-normal distribution: we expect effects in one direction with smaller effects more likely than larger ones (see Dienes, 2014). The prior (null) hypothesis has a mean of 0 (effect size of 0 between group means) with a standard deviation of 0.5, based on the mean difference observed by Chilosi et al. (2014).

**Fig. 4:**
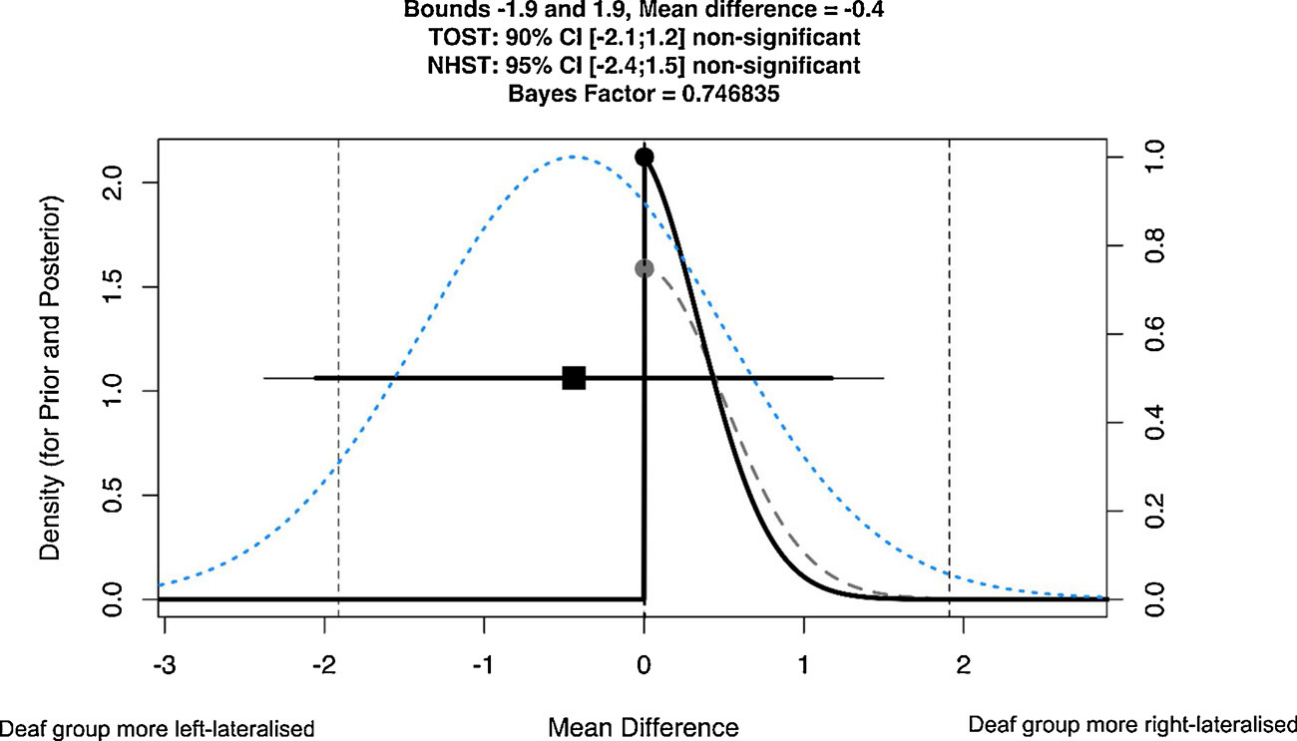
Plot showing the summary of equivalence testing supporting the interpretation that there is no meaningful difference in strength of LI during language production between the deaf and hearing children. The filled square indicates the mean difference of -0.4 with 90% confidence interval (CIs) as a bold horizontal line and 95% confidence intervals as a thin horizontal line. Equivalence bounds (-1.9 and 1.9 in raw units) are shown with vertical dashed lines. The light blue dotted line is the likelihood distribution, the light grey dashed line is the alternative distribution, modelled as a half-normal in light of hypotheses about the direction of the expected effect. This places less weight on observations in the unexpected direction when calculating the posterior likelihood function (solid black line) (For interpretation of the references to colour in this figure legend, the reader is referred to the web version of this article).

Fig. 4 shows the results of the two tests. Since the lower CI crosses the lower equivalence bound we cannot reject the possibility that the deaf group are more left-lateralised than the hearing group. However, a difference in this direction would not be predicted based on any of the previous literature. The lower 90% CI did not cross the upper bound, therefore we can reject the possibility that the deaf children are more right lateralised than the hearing children. In summary, this analysis supports the interpretation that there is no meaningful difference in strength of LI during language production between the deaf and hearing children. In addition, the Bayes factor of 0.746 provides moderate evidence for the null hypothesis (1.5 times more likely than the alternative hypothesis).

## 4 Discussion

The current study explored the effect of atypical sensory experience on hemispheric specialisation for language processing. Specifically, we assessed hemispheric lateralisation for language production in children born deaf. These children have great variability in their language experience and extremely limited auditory speech input. We used fTCD to measure hemispheric lateralisation of cerebral blood flow while the children generated language in response to an established Animation Description task (Bishop et al., 2009). As a group the deaf children showed left lateralisation whilst producing speech, sign, sign-supported English, or a combination of languages. This is the first study to measure cerebrovascular activity during language processing in a group of deaf children from a range of language backgrounds.

Due to the hypothesised link between atypical or weak lateralisation and language disorder (see Bishop, 2012 for a review), we chose a comparison group of hearing children matched on language ability. There was no significant difference between the deaf and hearing children in the strength of lateralisation during language production.

We chose to match deaf and hearing children on language level, as opposed to chronological age, to circumvent the potential confound of language proficiency on lateralisation. Specifically, a finding of reduced left-lateralisation in deaf compared to hearing children of the same age could be attributed to poorer language ability in the deaf group (consistent with a learning driven account) or to the difference in auditory input between groups (interpreted primarily as evidence for a signal driven account). It could be argued that a chronological age matched group would be required to mitigate against group differences associated with age-driven changes in lateralisation. However, two insights led us not to include a chronological age-matched group. The first is a meta-analysis of studies which examined age-related changes in language lateralisation. The authors suggest that the localised changes in functional lateralisation focused around the inferior frontal gyrus (within the range of the MCA and therefore of fTCD) are likely to reflect improved task performance rather than maturation per se (Weiss-Croft and Baldeweg, 2015). Indeed, language skills that have a more protracted period of skill development are most likely to display ‘age-related’ changes in lateralisation (Holland et al., 2007). The animation description task was designed such that very young children are able to complete it (Bishop et al., 2009). Selecting a task that taps early developing skills therefore reduces the likelihood of age-related effects between groups of different ages. The second insight is that two large studies using fTCD with children did not find an effect of age on lateralisation of language (Groen et al., 2012, Bishop et al., 2014). This suggests that should any subtle, age-related differences in lateralisation be present between groups, fTCD is not likely to be sensitive to them.

Our results agree with those of Chilosi et al. (2014), who reported left lateralisation for language production in a group of deaf children with CI. However, given that some participants in the current study had a CI and some did not, our interpretation of left lateralisation in deaf children is different to that of Chilosi et al. (2014). They argued, in line with an auditory input-driven theory, that ‘reafferation of auditory cortex following implantation’ (Chilosi et al., p. 4) drives left lateralisation. Their argument suggests that plasticity in response to cochlear implantation and subsequent input of speech to auditory cortices had led to left-lateralisation. The implication is that lateralisation was not present pre-implant, though crucially this was not explicitly tested. Furthermore, the Chilosi et al. (2014) study only included children with cochlear implants. Deaf children, with or without implants, access language through visual speech and, in some cases, a signed language: both of which have been shown to be robustly left-lateralised in deaf adults (see MacSweeney et al., 2008). Attributing left lateralisation of speech production in deaf children with CI to increased auditory input may therefore be premature. Indeed, our exploratory comparison between those with and without cochlear implants showed no significant difference between groups.

The two other viewpoints that have been put forward to account for language lateralisation, described in Section 1, suggest that linguistic processes or learning may drive lateralisation (see Minagawa-Kawai et al., 2011). While both accounts predict left lateralisation in deaf children producing (signed or spoken) language, only a ‘learning-based’ account would implicate a relationship with language proficiency. Specifically, a learning-driven account would predict that left-lateralised language correlates positively with measures of language proficiency. In the current study, we did not find evidence of a positive correlation between lateralisation and language ability either when the data from all children were pooled or when the data from deaf and hearing children were analysed separately. It is entirely possible that the measures used to assess language proficiency were not an accurate reflection of a child’s ability. This is a particularly problematic area in language research with deaf children, because of the lack of standardised measures to assess modes of communication such as sign supported English. Nevertheless, the offline measures of BSL comprehension, as well as reading and vocabulary were related to how much communication the deaf child attempted during the production period (r’s > 0.6). This suggests an acceptable level of validity for the measures of proficiency.

Whilst acknowledging that strong inferences cannot be drawn from a null result, the current data suggest that left-lateralisation is not conditional upon ‘successful’ language learning. This finding is in contrast to an fMRI study with adults showing increasing lateralisation with learning (Plante et al., 2015). Their study showed strengthening left-lateralisation of brain activity in hearing adults learning to discriminate words from pseudowords in an unknown language. Learning-related changes were reported in a region of interest encompassing the anterior and middle parts of the superior temporal gyrus. Assessing changes in lateralisation during learning, as opposed to correlations with overall proficiency, as was done in the current study, is a more direct way of testing the hypothesis that learning drives lateralisation. However, the types of processes involved in learning to discriminate words from non-words in an unfamiliar language are likely to be drastically different from those involved in producing novel sentences, making it difficult to meaningfully compare these studies. Furthermore, by studying hearing adults, Plante et al. (2015) contribute to a different question about the role of learning in driving lateralisation. Critically, hearing adults learning a second language already have established a robust first language. Changes in lateralisation may relate to the modification of an existing lateralised system. It is unlikely that conclusions regarding the impact of learning on hemispheric lateralisation drawn from adult second language learners can be applied to children learning a first language, or even children learning a second language (McNealy et al., 2011). We suggest that the trajectory of novel language lateralisation in hearing adults cannot afford insight into the weighting of maturation and experience in the development of lateralisation, since the two are inexorably linked in all but extreme cases (e.g. of neglect). In contrast, many of the deaf children tested in the current study do not have a robust first language and are therefore a more suitable population for answering questions about the role of learning in the development of lateralisation, distinct from effects of maturation. Using a longitudinal design to map the trajectory of language lateralisation in children who do not have experience of a rich language input from infancy would allow stronger inferences to be drawn, particularly about direction of causality. However, the data here do not provide evidence that proficient language learning relates to the strength of left hemisphere dominance.

Our data are most supportive of ‘domain-driven’ theories of lateralisation. That is, the data support the hypothesis that hemispheric lateralisation for language production is driven by the requirements of processing linguistic information regardless of modality, rather than auditory language experience. This concords with functional neuroimaging findings from deaf adults showing left-lateralised processing of various aspects of sign language processing (Corina et al., 2003; MacSweeney et al., 2002; Emmorey et al., 2002a).

All of the children in the current study, including those using predominantly BSL, are regularly exposed to spoken English via the visual modality and via whatever access to auditory speech is available via their hearing aid or CI. Therefore, a possible caveat to our support for a domain-driven account is that some low-level properties present in the auditory speech stream are perceptible from visual speech. Nevertheless, our data make clear that increased access to *auditory* speech via a CI is not a prerequisite for left hemispheric lateralisation for language production in deaf children.

Several extraneous factors may have influenced lateralisation in the current study. First, the animation clips contained environmental sounds. This introduces potential bias during the baseline period if perception of such sounds is lateralised in the hearing children. However, evidence for the lateralisation of non-speech sounds is equivocal (e.g. Boemio et al., 2005; Rosen et al., 2011). Moreover, there were no significant differences in lateralisation between deaf and hearing groups in LIs before baseline correction, (hearing mean = -0.52 (0.77), deaf mean = -.69 (1.1.), t (36) = -.56, p = .58, d = .18). This suggests that the environmental sounds from the video did not introduce significant group bias.

Another factor that requires consideration is that we assessed children on handedness preference but not handedness skill, which has been linked to brain asymmetry in a genome-wide association study meta-analysis (Brandler et al., 2013). Whether deaf and hearing children show systematic differences in handedness skill is an open question (see meta-analysis by Papadatou-Pastou and Sáfár, 2016), therefore we cannot rule out the possibility that differences in handedness skill between groups may have obfuscated differences in lateralisation. The issue of handedness is related to the final limitation raised here: the possibility of a contribution of motor movement to lateralisation in children producing BSL signs. Previous studies with signing adults have observed stronger lateralisation for sign than speech production, potentially linked to preparatory premotor activity (Gutierrez-Sigut et al., 2015a, b). In the subgroup of children who produced signs in their output (n = 6) we did not observe an association between the strength of signing hand dominance and lateralisation (−0.16, p = .79). However, given the very small group, this lack of relationship must be interpreted with caution.

In conclusion, this study provides a unique insight into the effect of drastically different language experience on hemispheric specialisation. It provides evidence that hemispheric specialisation for language production is robust to significant differences in language experience. This study also demonstrates the feasibility of using fTCD as an indirect measure of neural activity in this understudied paediatric population.

Funding: This work was funded by a Wellcome Trust Fellowship to M.M. (100229/Z/12/Z) and the Economic and Social Research Council (Deafness Cognition and Language Research Centre; RES-620-28-0002). EG was funded by the Spanish Ministerio de Economía y Competitividad (Grant Number PSI2014- 60611-JIN).

## 5 Declarations of interest

None.

## 6 Conflict of interest

We wish to confirm that there are no known conflicts of interest associated with this publication and there has been no significant financial support for this work that could have influenced its outcome.
